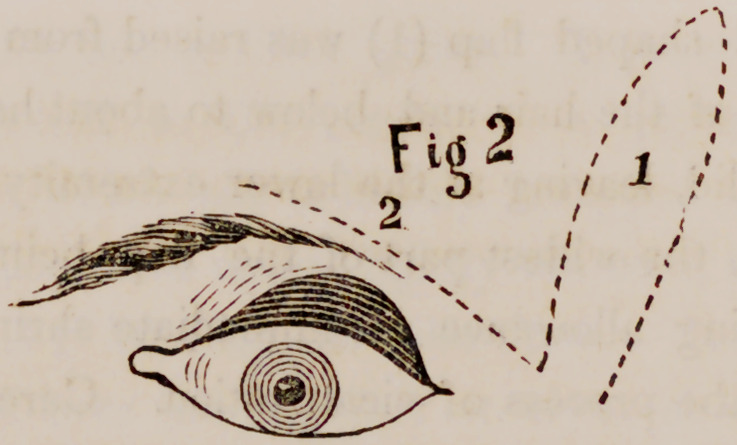# Plastic Operations

**Published:** 1848-11

**Authors:** 


ART. II.—*Plastic Operations.* By Dr. HAMILTON.—*Case* 1. *Ectropion of Lower Lid, and Restoration by Transplantation.*

*March* 18, 1848; operated upon Miss ———, of Lancaster, for an eversion of the left lower lid, produced by a burn, when an infant. Miss H. is now about eighteen years old. The eversion is to the extent of a little more than half an inch in its vertical diameter, and the whole breadth of the lid, in its transverse diameter.

About two years since a physician had operated by making a single longitudinal incision under the lid and then attempting to keep it open by various means; but this had resulted as all such attempts invariably do, in a final closure of the wound, and a return of the lid to the same condition in which it was before the operation.

Having brought the patient partially under the influence of chloroform, I operated in the following manner:

Fig 1

First an elliptical shaped flap (1) was raised from the temple, reaching above to the edge of the hair and below to about half an inch below the outer angle of the lid, leaving at the lower extremity a vinculum of about six lines in breadth, the widest part of the flap being about nine lines in breadth; thus making allowance for immediate shrinking and subsequent contraction during the process of cicatrization. Care was also taken to give sufficient thickness to the flap, and not to sharpen the upper end too much, from a neglect of which latter precaution I have sometimes lost the extreme end of the flap. An incision (2) was now made, commencing at the median or inner border of the lower extremity of the wound, and extending inward and upward to terminate at a point opposite the inner canthus, and about four lines below it; the middle of the incision having approached within about two lines of the lower lid; in its course upwards and inwards, the line of this incision made a slight convexity downwards. The knife was carried deep, and then by cutting upwards the lid was undermined and loosened.

Hemorrhage having nearly ceased, the flap was slid around and made to occupy the gaping wound made by incision (2), and here it was secured by a number of small sutures and by a compress and roller. The wound in the temple was now closed by sutures and straps.

The flap united in a few days without any loss of structure; and about three weeks after the first operation I cut off the vinculum, the wound from which soon closed. The wound in the temple closed very slowly.

The result of the operation has been a perfect restoration of the lid to its natural position, and the consequent removal of the deformity.

Some months after this patient had returned home, she was attacked with a peritonitis, at least so I infer from the accounts which I have received, and soon died. Her death had no connection, near or remote with the operation.

*Case* 2. *Ectropion of Upper Lid and Restoration by Transplantation.*

*March*, 1848; I operated upon Miss ———, of Buffalo, aged 16, for an eversion of the upper lid, occasioned by a burn when a child. Two or three years before the cicatrix had been divided, and an attempt made to keep the lid down during the reunion of the wound, but with the same result as in the case just described; the eversion soon became as complete as ever.

I operated by making a flap from the temple (1) and laying it by tortion into the incision (2), and closing it down with fine sutures. The wound in the temple was also closed by sutures and adhesive plasters. The flap united in a short time, except about three lines of its extremity, which sloughed either from undue pressure made upon it by a strap, or from its being too thin and therefore illy supplied with blood. The wound upon the temple healed slowly.

The result of the operation is, that the eversion and lifting of the lid is remedied so far as the flap was saved, but opposite the end of the flap where sloughing occurred, the eversion is reproduced.

The mode of operation adopted in the above cases is especially applicable where the eversion, as in both instances it was, is extreme; since by it alone can the restoration be completely effected. But care must be taken to have a sufficient vinculum, a thick flap and rounded at its end; all this to ensure its vitality. Care must be taken also that the flap should be considerably longer and wider than the loss to be supplied, lest by contraction it should become too small. The wound made to receive the flap ought to be deep, lest the new skin should pout, and it must be kept in place by fine stitches and *moderate pressure.*

I have lately seen a case in which the flap has gradually thickened and finally assumed the appearance of a tumor, of about the size of a hazlenut.

The wound in the temple will hereafter be made to unite more promptly and effectually by the use of the new adhesive plaster.

Where the eversion is less, the older method will still be preferred, as we have often demonstrated, and as the following case will show.

*Case* 3. *Ectropion and Operation by Excision of a Triangular Piece.* Mr. ———, of Ohio, with an eversion of lower lid to a moderate extent; the consequence of a burn in early life. In this case the operation of cutting through the cicatrix had been *twice* made, and with no better results than in each of the above cases.

I operated by cutting out a triangular piece in the centre of the tarsus, the base of the triangle corresponding to the edge of the tarsus; I *then* cut up the attachments below, and drew the base of the triangle together with a small suture.

The result is an almost complete restoration of the lid to its natural position.

## Figures and Tables

**Fig. 1 f1:**
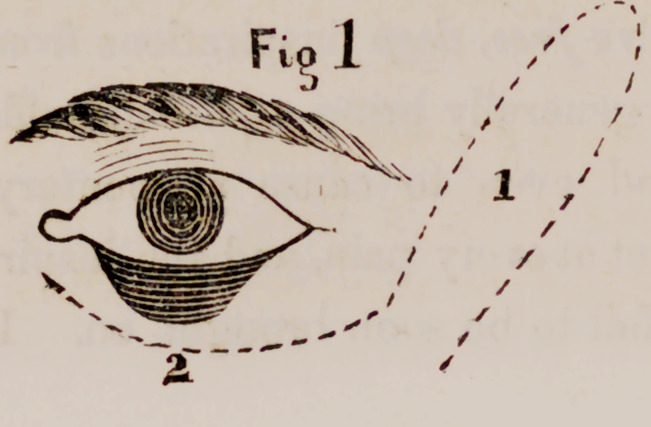


**Fig. 2 f2:**